# Subjective cognitive decline may mediate the occurrence of postoperative delirium by P-tau undergoing total hip replacement: The PNDABLE study

**DOI:** 10.3389/fnagi.2022.978297

**Published:** 2022-11-30

**Authors:** Fanghao Liu, Xu Lin, Yanan Lin, Xiyuan Deng, Rui Dong, Bin Wang, Yanlin Bi

**Affiliations:** ^1^Department of Anesthesiology, Qingdao Municipal Hospital Affiliated to Qingdao University, Qingdao, China; ^2^Department of Anesthesiology, Nanjing Drum Tower Hospital, Nanjing, China

**Keywords:** subjective cognitive decline, postoperative delirium, total hip replacement, cerebrospinal fluid biomarkers, mediation effect

## Abstract

**Objective:**

We again investigated the relationship between subjective cognitive decline (SCD) and postoperative delirium (POD) with a larger sample queue. We also determined whether SCD could cause the occurrence of POD through cerebrospinal fluid (CSF) biomarkers.

**Methods:**

A prospective, observational cohort study was implemented in the Qingdao Municipal Hospital Affiliated with Qingdao University. This study recruited 1,471 qualified patients affiliated with the Perioperative Neurocognitive Disorder And Biomarker Lifestyle (PNDABLE) study scheduled for total hip replacement under combined spinal and epidural anesthesia from June 2020 to May 2022. The Mini-Mental State Examination (MMSE) and the Montreal Cognitive Assessment (MoCA) were used to assess the cognitive level of the patients the day before surgery. Pittsburgh sleeps quality index (PSQI) scale was used to assess sleep status. Patients were divided into the SCD group and the non-SCD (NSCD) group based on the Subjective Cognitive Decline Scale (SCDS). CSF was collected after a successful spinal-epidural combined puncture, and amyloid-β_40_ (Aβ_40_), amyloid-β_42_ (Aβ_42_), total tau (T-tau), and phosphorylated tau (P-Tau) in CSF were analyzed by enzyme-linked immunosorbent assays. After the surgery, the incidence of POD was determined by the Confusion Assessment Scale (CAM), and Memorial Delirium Assessment Scale (MDAS) score was used to determine the severity of POD. Logistic regression and sensitivity analyses were performed to determine the relationship between CSF biomarkers, SCD, and POD. The mediating effect was used to analyze the function of specific CSF biomarkers in the relationship between SCD and POD. The risk factors of SCD were also separately verified by logistic regression and sensitivity analysis models.

**Results:**

The total incidence rate of POD was 19.60% (*n* = 225/1148), which was 29.3% (*n* = 120/409) in the SCD group and 14.2% (*n* = 105/739) in the NSCD group. We comprehensively considered the effect of covariates such as age, hypertension, and diabetes. Multivariate logistic regression analysis showed that SCD (OR = 1.467, 95%CI: 1.015–2.120, *p* = 0.042) and P-tau (OR = 1.046, 95%CI: 1.028–1.063, *p* < 0.001) were risk factors for POD. The sensitivity analysis results were consistent with the above results. Mediation analysis showed that the relationship between SCD and POD was partially mediated by P-tau, which accounted for 31.25% (P-tau, IE = 4.279 × 10^−2^, *p* < 0.001). For SCD, the results of logistic regression analysis models showed that age (OR = 1.035, 95% CI: 1.020–1.049, *p* < 0.001), higher preoperative PSQI score (OR = 1.047, 95%CI: 1.014–1.080, *p* = 0.005), and P-tau (OR = 1.015, 95%CI: 1.002–1.028, *p* = 0.021) were risk factors for SCD, and subsequent sensitivity analysis confirmed this result after adjustment for ASA grade, height, and weight.

**Conclusion:**

Patients with SCD are more likely to develop POD undergoing total hip replacement, and SCD can mediate the occurrence of POD *via* P-tau.

**Clinical trial registration:**

This study was registered at China Clinical Trial Registry (Chictr2000033439).

## Introduction

Postoperative delirium (POD) is an acute temporary brain dysfunction after surgery. It is characterized by attention, consciousness, and cognitive impairment in the patient and often fluctuates within a short time (American Psychiatric Association, 2013). With the proposal of the new concept of perioperative neurocognitive disorders (PND), the classification and definition of perioperative cognitive function changes were also updated. As one of its subclasses, POD refers to delirium occurring within 7 days after surgery or before discharge (Evered et al., 2018). The incidence of POD reported by different studies varied greatly, with a basic fluctuation of 17–61% in major surgeries, which can be closely associated with the subject population, the diagnosis method, and medical intervention (de Lange et al., 2013; Janssen et al., 2019). The general view is that POD is the result of multiple factors; however, its pathogenesis is still unclear (Maldonado, 2013, 2018; Wang and Shen, 2018). Studies suggest a reciprocal relationship between the amyloid-beta (Aβ), tau, and cognitive decline (Bateman et al., 2012; Scheltens et al., 2016). Soluble amyloid-β_42_ (Aβ_42_) chronic combination with neurons through the blood–brain barrier (BBB) can destroy brain homeostasis, damage the function of neurons, and lead to the occurrence of POD (Acharya et al., 2015). On the other hand, Low and medium Aβ_42_ levels along with high Tau levels in cerebrospinal fluid (CSF) can predict the occurrence of neurodegenerative diseases (Tapiola et al., 2009). The role of these proteins in the mechanism of POD is yet to be determined.

Subjective cognitive decline (SCD) is a condition of cognitive decline in self-cognition. No validation of the subjective experience of a cognitive complaint by abnormalities on objective neuropsychological assessments is required conceptually (Jessen et al., 2014). SCD is an unspecific syndrome with multiple possible underlying etiologies, its existence may accelerate the descent of cognitive level (Gallassi et al., 2010; Jessen et al., 2014). Presently, even though the discussion of SCD is more focused on a prodromal stage of Alzheimer’s disease (AD), SCD possibly serves more appropriately as a transition zone between the cognitive integrity phase and numerous potential cognitive deficits, including mild cognitive impairment (MCI) and various dementias(Cheng et al., 2017; Viviano and Damoiseaux, 2020). In recent years, many CSF biomarkers were introduced in the guideline of AD diagnosis, such as Aβ_42_, total tau (T-tau), and phosphorylated tau (P-tau), and their fluctuation concentration is also conducive to predicting cognitive decline(McKhann et al., 2011; Mutlu et al., 2017). POD is considered to present with identical or similar CSF-based biomarkers, similar to AD. It can be presumed that SCD is prospectively associated with underlying POD pathogenesis.

In this study, we aimed to determine the relationship between SCD and POD based on CSF biomarkers. Compared with the previous phased study (Lin et al., 2021), this study included more patients who fulfilled the inclusion criteria. In addition to determining the relationship among CSF biomarkers (Aβ_42_, Aβ_40_, T-tau, P-tau), SCD, and POD, we elucidated the internal mechanisms and the effect of SCD, CSF biomarkers, and POD, and studied the risk factors of SCD in depth.

## Materials and methods

### PNDABLE study

All participants recruited in this study were a part of the Perioperative Neurocognitive Disorder And Biomarker Lifestyle (PNDABLE) study. PNDABLE study is an underway, single-center, and large population-based cohort study started in 2018 at the Qingdao Municipal Hospital in Shandong Province, China. We aimed to determine the risk factors and pathological mechanism of PND in the northern Chinese Han population by performing comprehensive clinical and neuropsychological tests and extracting and analyzing biomarkers from the peripheral blood and CSF samples of enrolled patients. We aimed to find a new path for its early prophylaxis and treatment. PNDABLE study was approved by the Ethics Committee of Qingdao Municipal Hospital affiliated with Qingdao University (Ethical Committee N°2020 *PRO FORMA* Y number 005) and registered in the Chinese Clinical Trial Registry (Registration number: CHICTR2000033439). Written informed consent was received from all the patients before participating in the study.

### Participants

In this study, we recruited 1,471 qualified patients scheduled for total hip replacement under combined spinal and epidural anesthesia from June 2020 to May 2022 in the Qingdao Municipal Hospital affiliated with Qingdao University. The inclusion criteria were as follows: (1) age 40–90 years, (2) Han population of northern China, (3) American Society of Anesthesiologists (ASA) Grade I–II, (4) intact preoperative cognitive function without language communicative disability, and (5) level of education was sufficient to co-operate preoperative cognitive function tests. Exclusion criteria for this study were as follows: (1) preoperative delirium, head trauma, epilepsy, dementia caused by diverse reasons, or other major psychological dysfunction, (2) severe psychiatric mental illness, such as depression, anxiety, obsessive–compulsive disorder, and even schizophrenia, (3) preoperative MMSE score not more than 23 or MOCA score not more than 26, (4) severe systemic diseases that may affect CSF or blood-related biomarkers, mainly including malignant tumors and central nervous system infection, (5) familial genetic diseases, and (6) unwillingness to comply with the protocol or procedures.

### Anesthesia and surgery

All patients strictly complied with at least 6-h fasting and 2-h water fasting without any preoperative medication before the surgery. First, the efficient vascular passage was established rapidly after verifying the identities of patients after entering the operation room. Oxygen saturation (SpO_2_), non-invasive arterial blood pressure (NABP), heart rate (HR), and electrocardiogram (ECG) were monitored in real-time throughout the surgery. The spinal and epidural anesthesia was administered in the lateral position on the L_3–4_ space. After a successful puncture, 2 ml of CSF was immediately extracted by a 5 ml syringe, and 0.67% ropivacaine 2.0–2.5 ml was injected into the subarachnoid space. An appropriate amount of 2% lidocaine was injected into the epidural catheter when needed for maintaining the level of anesthesia at T_10_.

The surgery was performed in the lateral position. The conventional surgery area was disinfected and covered with sterile surgical towels. Gibson incision was made and then the damaged femoral head and acetabulum were cleaned, shaped, and carefully embedded in an artificial acetabulum cup. The suitable hip prosthesis was then inserted and monitored by X-ray. Finally, rinsed the surgical incision, and the skin incision was closed.

After the surgery, patients were closely monitored in the post-anesthetic ICU (PACU) room and were sent to the ward until the recovery of sensory and motor skills. All surgeries were performed by the same surgical team to avoid corresponding bias, and the surgery time, anesthesia time, infusion volume, bleeding volume, and urine volume during the perioperative period were recorded.

All patients received a postoperatively patient-controlled intravenous analgesia (PCIA) for 48 h, which contained 2.5 μg·kg^−1^ sufentanil, 5 mg tropisetron, and 0.9% normal saline (total volume: 100 ml, basal rate: 2 ml/h, additional volume: 0.5 ml/time, lockout time: 15 min). The numerical rating scale (NRS) was used for the evaluation of pain release after removing PCIA (Hjermstad et al., 2011).

### Collections and measurements of CSF samples

The CSF samples (2 ml) were processed immediately within 2 h after the spinal and epidural anesthesia. Each sample was centrifuged immediately at 2,000 *g* for 10 min at room temperature, then separated and stored in an enzyme-free Eppendorf tube (AXYGEN; PCR-02-C) at −80°C, which can undergo two freeze–thaw cycles. The levels of Aβ_40_ (BioVendor, Ghent, Belgium Lot: No. 292–6,230), Aβ_42_ (BioVendor, Ghent, Belgium Lot: No. 296–64,401), T-tau (BioVendor, Ghent, Belgium Lot: No. EK-H12242), and P-tau (BioVendor, Ghent, Belgium Lot: QY-PF9092) in CSF were determined by enzyme-linked immunosorbent assay (ELISA) using INNOTEST (Fujirebio Europe N.V.) on the microplate reader (Thermo Scientific MultiskanMK3). All ELISA measurements were performed by well-experienced technicians according to the instructions of the kit. Each sample was measured in duplicate, and the average value of repeated measurement was used for statistical analysis. All antibodies and microplates belonged to the same batch (the CV of inter-assay <5% and the CV of intra-assay <15%) to exclude corresponding bias.

### Cognitive assessments

The Mini-Mental State Examination (MMSE) and the Montreal Cognitive Assessment (MoCA) were performed to assess the cognitive level of the patients the day before surgery. Regarding the investigation of SCD, a gold standard for the diagnosis of SCD was not yet available. Nonetheless, to simply assess and quantify SCD, we designed and used the Subjective Cognitive Decline Scale (SCDS) to diagnose SCD in a preoperative interview, and participants were divided into the SCD group and the non-SCD (NSCD) group based on their symptoms. SCDS was designed to focus on the memory function of the respondents based on SCD-I and the hospital environment was taken as the studying background, which regulated the following two evaluation methods: classification index (Part I) and continuous index (Part III), set Part II as a supplement according to SCD-plus criteria simultaneously(Jessen et al., 2014; Molinuevo et al., 2017). The diagnostic criteria for SCD were as follows: (1) compared with the past, the patient’s memory decreased significantly, which was considered to be the basic condition for the diagnosis of SCD. (2) The scores of MMSE and MoCA were normal and excluded abnormalities caused by other neuropsychiatric diseases (except Alzheimer’s disease), medical disorders, drugs, or other substances abused (Jorm et al., 1997; Rundshagen, 2014; Molinuevo et al., 2017). Meanwhile, the Pittsburgh Sleep Quality Index (PSQI) scale was used to assess the sleep status of the patients (The total score range was 0–21. The lower the score, the better the sleep status; Baglioni et al., 2016).

The occurrence of POD was assessed by the Confusion Assessment Method (CAM) at 10 a.m. and 2 p.m. twice a day by an anesthesiologist for 1–7 days (or before discharge; Leung et al., 2008). The diagnosis of POD includes the following four clinical criteria: (1) acute attack and fluctuation process, (2) inattentiveness, (3) confusion of thinking, and (4) change of consciousness level. POD was diagnosed if it fulfilled (1) and (2) and (3) or (4) at the same time. Furthermore, Memorial Delirium Assessment Scale (MDAS) score reflected the severity of POD (Schuurmans et al., 2003).

Neurologists participated in all preoperative neuropsychological tests for patients. Correspondingly, postoperative visits and measurements were performed by specialized anesthesiologists. None of the researchers in charge of cognitive evaluation were involved in other relevant parts of the study, and they received a two-week unified training before this procedure.

### Statistical analysis

Comprehensively, considering the implementation conditions and logistic regression analysis of 20 covariates in this study, POD incidence was 17% (Oh et al., 2021), and assuming a follow-up rate loss of 20%, the required sample size was calculated as 1,471 cases (20 × 10 ÷ 0.17 ÷ 0.8 = 1,471).

SPSS version 21.0 (SPSS, Inc., Chicago, IL, United States) and STATA/MP version 16.0 (StataCorp, College Station, Texas) were used for statistical analysis. For continuous data, the normality and homogeneity of variance were calculated by Shapiro–Wilk test and F-test, respectively. The preoperative baseline status and intraoperative data of patients were compared by performing a T-test or Chi-Square test. Similarly, the T-test was performed to compare the CSF biomarker groups. Then, with the NSCD group as a reference, the associations of SCD and CSF biomarkers with POD and SCD risk factors were further verified using univariate and multivariate logistic regression models, respectively. Additionally, a sensitivity analysis was performed to assess result stability. Finally, to explore whether the specific CSF biomarkers selected from the logistic regression models could mediate the relationship between SCD and POD, mediation analyses were fitted based on a generalized structural equation model. Promotion >10% was regarded to have a significant mediation effect. Among all statistical models, *p* < 0.05 was considered significant. All statistical methods have been reviewed by professional statistician.

## Results

### Participant characteristics

**Figure 1:**
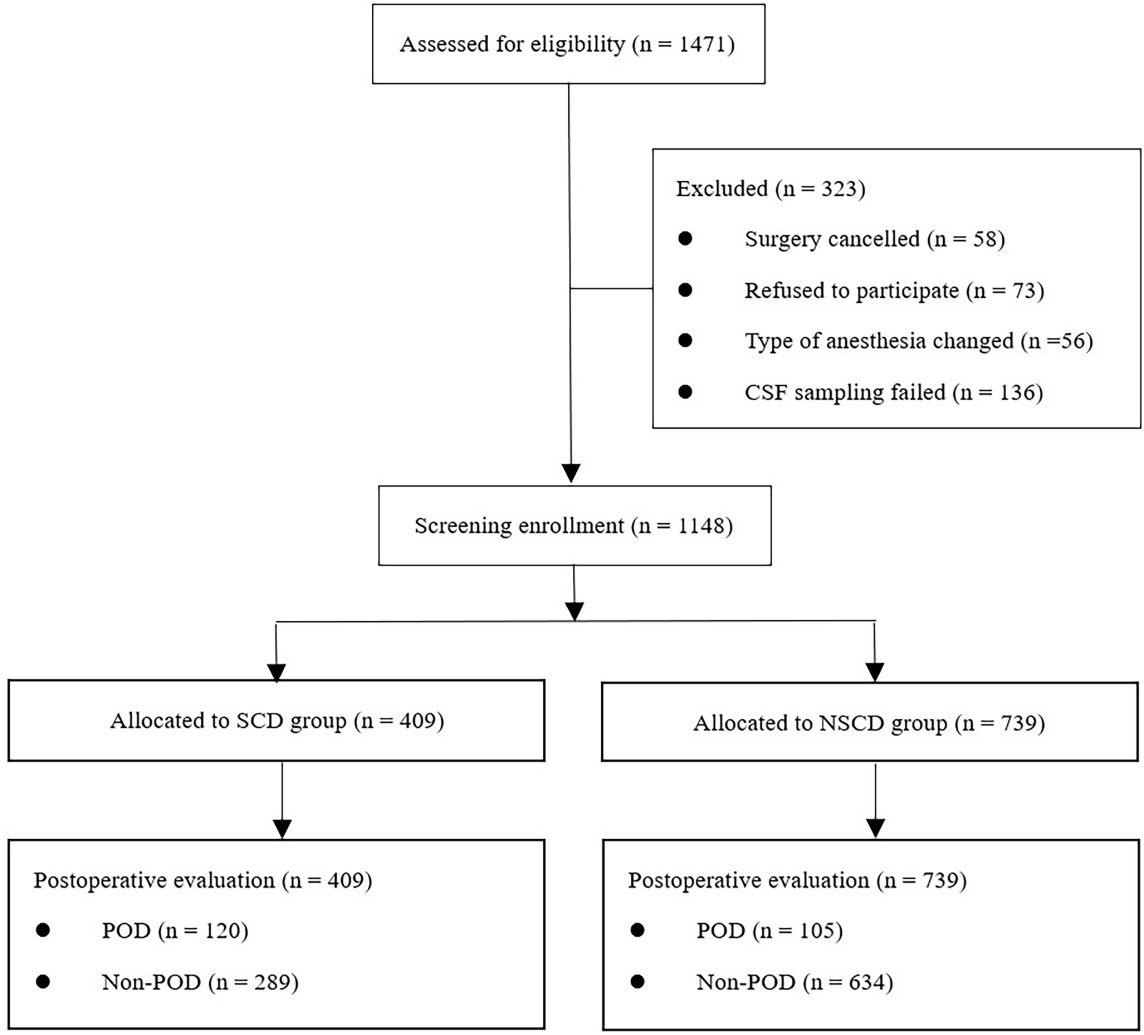
Flowchart depicting the inclusion and exclusion criteria.

As of May 2022, 1,471 eligible participants were enrolled in the study; 1,148 of these were included in the final statistical analysis. A total of 409 and 739 participants were divided into the SCD and NSCD groups, respectively, according to SCDS evaluation results. The flowchart of inclusion and exclusion criteria is presented in Figure 1.

As shown in Table 1, the incidence rate of POD in the SCD group was 29.3% (*n* = 120/409), which was significantly higher than the incidence rate of 14.2% (*n* = 105/739) in the NSCD group (*p* < 0.001). The model grouped by age stages suggested that SCD incidence was significantly different among different groups (*p* < 0.001). Similar differences were also observed in the model grouped according to education years (*p* < 0.05). Compared with the NSCD group, preoperative PSQI scores were higher in patients with SCD (*p* < 0.05). Nevertheless, no significant difference regarding other demographic and clinical characteristics was observed between the SCD and NSCD groups (*p* > 0.05).

**Table 1:** Demographic and clinical characteristics of the study participants.

	SCD Group	NSCD Group	*p*-value
	(n = 409)	(n = 739)	
Age (year,x̄ ± s)			< 0.001
40 ~ 49, n (%)	3 (0.7%)	15 (2.0%)	
50 ~ 59, n (%)	104 (25.4%)	284 (38.4%)	
60 ~ 69, n (%)	150 (36.7%)	276 (37.3%)	
70 ~ 79, n (%)	107 (26.2%)	133 (18.0%)	
80 ~ 90, n (%)	45 (4.0%)	31 (4.2%)	
Sex, n (%)			0.694
Male, n (%)	212 (51.8%)	392 (53.0%)	
Female, n (%)	197 (48.2%)	347 (47.0%)	
Height (cm,x̄ ± s)	166.71 ± 7.44	167.44 ± 7.63	0.118
Body weight (kg,x̄ ± s)	70.59 ± 11.90	71.22 ± 12.28	0.401
ASA grade, n (%)			0.179
I, n (%)	267 (65.3%)	511 (69.1%)	
II, n (%)	142 (34.7%)	228 (30.9%)	
Hypertension n, (%)	155 (37.9%)	269 (36.4%)	0.615
Diabetes n, (%)	70 (17.1%)	108 (14.6%)	0.262
Coronary heart disease n, (%)	51 (12.5%)	74 (10.0%)	0.201
Drinking history n, (%)	113 (27.6%)	229 (31.0%)	0.233
Smoking history n, (%)	99 (24.2%)	203 (27.5%)	0.229
Years of education n, (%)			0.029
0 n, (%)	11 (2.7%)	22 (3.0%)	
1 to 6 n, (%)	93 (22.7%)	117 (15.8%)	
7 to 9 n, (%)	138 (33.7%)	265 (35.9%)	
10 to 12 n, (%)	83 (20.3%)	191 (25.8%)	
>12 n, (%)	84 (20.5%)	144 (19.5%)	
Preoperative MMSE score (point,x̄ ± s)	27.97 ± 1.64	28.11 ± 1.57	0.178
Preoperative MoCA score (point,x̄ ± s)	28.15 ± 0.79	28.15 ± 0.91	0.92
Preoperative PSQI score (point,x̄ ± s)	7.43 ± 3.87	6.72 ± 4.09	0.004
Infusion volume (ml,x̄ ± s)	852.69 ± 113.84	846.96 ± 99.39	0.375
Bleeding volume (ml,x̄ ± s)	121.15 ± 14.86	119.27 ± 16.24	0.053
Urine volume (ml,x̄ ± s)	217.52 ± 127.52	230.52 ± 128.00	0.099
Operation time (min,x̄ ± s)	122.05 ± 12.06	121.44 ± 12.26	0.415
Anesthesia time (min,x̄ ± s)	146.05 ± 14.05	145.12 ± 14.71	0.298
POD, n (%)	120 (29.3%)	105 (14.2%)	< 0.001
Postoperative MDAS score (point,x̄ ± s)	7.28 ± 6.85	6.54 ± 5.77	0.063
Postoperative 24 h NRS score (point,x̄ ± s)	1.99 ± 1.25	2.02 ± 1.28	0.644

### CSF biomarkers between the SCD and NSCD groups

When focusing on differences in the CSF biomarkers between the two groups, the independent sample T-test showed contrasts in the level and ratio of many indicators, including Aβ_42_, T-tau, P-tau, Aβ_40_/Aβ_42_, Aβ_40_/T-tau, Aβ_42_/T-tau, and Aβ_42_/P-tau (*p* < 0.05; Table 2).

**Table 2:** CSF biomarkers between the SCD group and NSCD group.

	SCD Group	NSCD Group	*p*-value
	(*n* = 409)	(*n* = 739)	
Aβ_40_ (pg / ml,x̄ ± s)	6230.85 ± 4092.10	6249.73 ± 4131.80	0.941
Aβ_42_ (pg / ml,x̄ ± s)	313.55 ± 206.78	340.49 ± 205.95	0.034
T-tau (pg / ml,x̄ ± s)	279.99 ± 185.22	229.19 ± 141.52	< 0.001
P-tau (pg / ml,x̄ ± s)	47.72 ± 21.53	41.20 ± 17.06	< 0.001
Aβ_40_ / Aβ_42_	28.47 ± 31.61	23.48 ± 19.50	0.004
T-tau / P-tau	6.25 ± 3.91	5.94 ± 3.56	0.192
Aβ_40_ / T-tau	27.74 ± 19.11	32.39 ± 23.62	< 0.001
Aβ_42_ / T-tau	1.47 ± 1.13	1.81 ± 1.33	< 0.001
Aβ_42_ / P-tau	7.99 ± 7.32	9.34 ± 6.93	0.002

### Relationships between CSF biomarkers, SCD, and POD

The results of the univariate logistic regression analyses showed that SCD (odds ratio (OR) = 2.507, 95% confidence interval (CI): 1.864 ~ 3.373, *p* < 0.001), T-tau (OR = 1.004, 95% CI: 1.003 ~ 1.005, *p* < 0.001), and P-tau (OR = 1.045, 95% CI: 1.037 ~ 1.054, *p* < 0.001) were risk factors for POD. In contrast, Aβ_42_ (OR = 0.998, 95% CI: 0.997 ~ 0.999, *p* < 0.001) was a protective factor for POD. After synthetically analyzing the perioperative factors including age, sex, hypertension, diabetes, coronary heart disease, drinking history, smoking history, education years, preoperative MMSE score, preoperative MoCA score, preoperative PSQI score, Aβ_40_, Aβ_42_, T-tau, P-tau, Aβ_40_/Aβ_42_, T-tau/P-tau, Aβ_40_/T-tau, Aβ_42_/T-tau, and Aβ_42_/P-tau, we performed multivariate regression analyses for POD. Only SCD (OR = 1.467, 95% CI: 1.015 ~ 2.120, *p* = 0.042) and P-tau (OR = 1.046, 95% CI: 1.028 ~ 1.063, *p* < 0.001) were still considered risk factors for POD. Based on these results, we included more covariates such as sex, ASA grade, height, weight, drinking history, smoking history, education years, infusion volume, bleeding volume, and urine volume to perform further sensitivity analysis, and the results were consistent with those of the multivariate regression analysis [SCD (OR = 1.457, 95% CI: 1.004 ~ 2.116, *p* = 0.048), P-tau (OR = 1.044, 95% CI: 1.027 ~ 1.062, *p* < 0.001)], showing result stability (Table 3).

**Table 3:** Logistic regression analysis of the influencing factors of POD.

	Unadjusted		Adjusted		Sensitivity analysis	
	OR (95%CI)	*p*-value	OR (95%CI)	*p*-value	OR (95%CI)	*p*-value
SCD	2.507 (1.864 ~ 3.373)	< 0.001	1.467 (1.015 ~ 2.120)	0.042	1.457 (1.004 ~ 2.116)	0.048
Aβ_40_	1.000 (1.000 ~ 1.000)	0.033	1.000 (1.000 ~ 1.000)	0.917	1.000 (1.004 ~ 1.000)	0.917
Aβ_42_	0.998 (0.997 ~ 0.999)	< 0.001	0.997 (0.995 ~ 1.000)	0.06	0.997 (0.995 ~ 1.000)	0.063
T-tau	1.004 (1.003 ~ 1.005)	< 0.001	1.000 (0.997 ~ 1.002)	0.766	1.000 (0.997 ~ 1.003)	0.945
P-tau	1.045 (1.037 ~ 1.054)	< 0.001	1.046 (1.028 ~ 1.063)	< 0.001	1.044 (1.027 ~ 1.062)	< 0.001

Multivariate logistic regression analysis models after adjusting for age, hypertension, diabetes, coronary heart disease, preoperative MMSE score, preoperative MoCA score, preoperative PSQI score, SCD, Aβ40, Aβ42, T-tau, P-tau, Aβ40/Aβ42, T-tau/P-tau, Aβ40/T-tau, Aβ42/T-tau, Aβ42/P-tau, operation time, anesthesia time, and postoperative 24-h NRS score.

Sensitivity analysis added more covariates, including sex, ASA grade, height, weight, drinking history, smoking history, years of education, infusion volume, bleeding volume, and urine volume.

**Figure 2:**
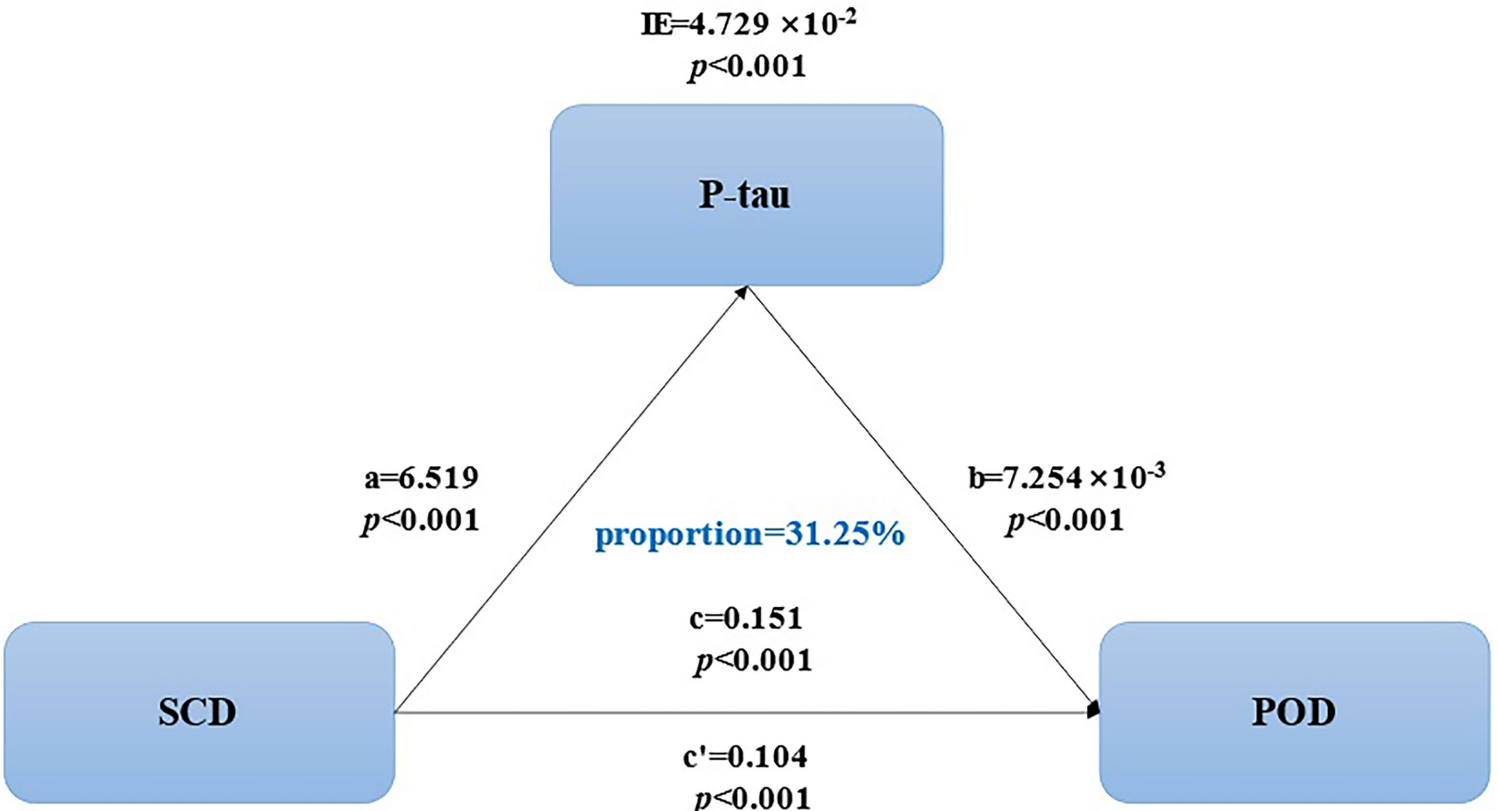
Mediation analyses of P-tau, SCD, and POD.

We further explored whether the specific CSF biomarkers selected from the logistic regression models could mediate the relationship between SCD and POD. The final mediating analysis showed that the relationship between SCD and POD was partially mediated by P-tau, with the approximate proportion of mediation being 31.25% (P-tau, IE = 4.279 × 10^−2^, *p* < 0.001; Figure 2).

### Factors affecting SCD

The same regression analysis models were established with SCD as a dependent variable as shown in Table 4. The univariate logistic regression analyses showed age (OR = 1.042, 95% CI: 1.029 ~ 1.055, *p* < 0.001), higher preoperative PSQI score (OR = 1.044, 95% CI: 1.014 ~ 1.076, *p* = 0.004), T-tau (OR = 1.002, 95% CI: 1.001 ~ 1.003, *p* < 0.001), and P-tau (OR = 1.018, 95% CI: 1.011 ~ 1.024, *p* < 0.001) as risk factors for SCD, whereas Aβ_42_ (OR = 0.999, 95% CI: 0.999 ~ 1.000, *p* = 0.035) was a protective factor for SCD. When multivariate logistic regression analysis was performed after adjusting for age, sex, hypertension, diabetes, coronary heart disease, drinking history, smoking history, education years, preoperative MMSE score, preoperative MoCA score, preoperative PSQI score, Aβ_40_, Aβ_42_, T-tau, P-tau, Aβ_40_/Aβ_42_, T-tau/P-tau, Aβ_40_/T-tau, Aβ_42_/T-tau, and Aβ_42_/P-tau, age (OR = 1.035, 95% CI: 1.020 ~ 1.049, *p* < 0.001), higher preoperative PSQI score (OR = 1.047, 95% CI: 1.014 ~ 1.080, *p* = 0.005), and P-tau (OR = 1.015, 95% CI: 1.002 ~ 1.028, *p* = 0.021) were still risk factors for SCD. The sensitivity analysis also showed almost similar results [age (OR = 1.034, 95% CI: 1.019 ~ 1.049, *p* < 0.001), higher preoperative PSQI score (OR = 1.046, 95% CI: 1.013 ~ 1.079, *p* = 0.006), and P-tau (OR = 1.016, 95% CI: 1.003 ~ 1.029, *p* = 0.017)], even after adding more covariates, including ASA grade, height, and weight.

**Table 4:** Logistic regression analysis of the influencing factors of SCD.

	Unadjusted		Adjusted		Sensitivity analysis	
	OR (95%CI)	*p*-value	OR (95%CI)	*p*-value	OR (95%CI)	*p*-value
Age	1.042 (1.029 ~ 1.055)	< 0.001	1.035(1.020 ~ 1.049)	< 0.001	1.034 (1.019 ~ 1.049)	< 0.001
Sex	0.953 (0.748 ~ 1.213)	0.694	1.107(0.823 ~ 1.487)	0.502	1.178 (0.847 ~ 1.639)	0.33
Hypertension	1.066 (0.831 ~ 1.369)	0.615	0.922 (0.702 ~ 1.211)	0.561	0.925 (0.700 ~ 1.221)	0.582
Diabetes	1.206 (0.869 ~ 1.675)	0.263	0.973 (0.676 ~ 1.402)	0.884	0.974 (0.676 ~ 1.403)	0.887
Coronary heart disease	1.280 (0.876 ~ 1.870)	0.202	1.097 (0.719 ~ 1.673)	0.668	1.106 (0.724 ~ 1.691)	0.641
Drinking history	0.850 (0.651 ~ 1.110)	0.234	0.883 (0.622 ~ 1.254)	0.488	0.892 (0.628 ~ 1.269)	0.526
Smoking history	0.843 (0.639 ~ 1.113)	0.229	0.897 (0.615 ~ 1.309)	0.575	0.904 (0.618 ~ 1.322)	0.603
Years of education	0.982 (0.953 ~ 1.012)	0.245	0.992 (0.958 ~ 1.028)	0.666	0.994 (0.959 ~ 1.030)	0.722
Preoperative MMSE score	0.949 (0.880 ~ 1.024)	0.178	1.035 (0.945 ~ 1.134)	0.46	1.034 (0.944 ~ 1.134)	0.47
Preoperative MoCA score	1.007 (0.877 ~ 1.156)	0.923	1.081 (0.931 ~ 1.255)	0.307	1.082 (0.931 ~ 1.256)	0.305
Preoperative PSQI score	1.044 (1.014 ~ 1.076)	0.004	1.047 (1.014 ~ 1.080)	0.005	1.046 (1.013 ~ 1.079)	0.006
Aβ_40_	1.000 (1.000 ~ 1.000)	0.941	1.000 (1.000 ~ 1.000)	0.114	1.000 (1.000 ~ 1.000)	0.11
Aβ_42_	0.999 (0.999 ~ 1.000)	0.035	1.001 (0.999 ~ 1.002)	0.505	1.001 (0.999 ~ 1.002)	0.493
T-tau	1.002 (1.001 ~ 1.003)	< 0.001	1.000 (0.998 ~ 1.002)	0.951	1.000 (0.998 ~ 1.002)	0.908
P-tau	1.018 (1.011 ~ 1.024)	< 0.001	1.015 (1.002 ~ 1.028)	0.021	1.016 (1.003 ~ 1.029)	0.017

Multivariate logistic regression analysis models after adjusting for age, sex, hypertension, diabetes, coronary heart disease, drinking history, smoking history, years of education, preoperative MMSE score, preoperative MoCA score, preoperative PSQI score, Aβ40, Aβ42, T-tau, P-tau, Aβ40/Aβ42, T-tau/P-tau, Aβ40/T-tau, Aβ42/T-tau, and Aβ42/P-tau.

Sensitivity analysis added more covariates, including ASA grade, height, and weight.

## Discussion

We expanded the conceptual framework of SCD to the field of PND *via* this study and investigated the potential relationship between SCD and POD for the first time. We had already found that SCD is one of the preoperative risk factors of POD in our pre-phase article (Lin et al., 2021). Including more eligible patients, we revalidated the results of the previous article in this study, as well as CSF biomarkers, were screened out as mediators to explore their intermediary roles in the relationship between SCD and POD.

POD is a neurobehavioral syndrome caused by multiple complex factors. Over the years, the association between MCI, a variety of neurodegenerative disorders, and postoperative cognitive dysfunction has been confirmed in diverse studies (Langa and Levine, 2014; Kirova et al., 2015). SCD is an intermediate state between cognitive normality and MCI, which is often cited as the first symptom of incipient neurodegenerative disease (Reisberg and Shulman, 2009). Studies correlating SCD with AD biomarkers had revealed that greater medial temporal lobe atrophy and cerebral hypometabolism in parietotemporal and parahippocampal regions have been found in patients with SCD(Amariglio et al., 2012; Wang et al., 2020), which resulted in reducing the tolerance of the brain to external harmful stimulations during the perioperative period. Few studies on the association between SCD and POD are available in cognitive psychology. The evaluation of the postoperative cognitive status of all participants by CAM showed that the total incidence of POD in all patients included in this study was 19.60% (*n* = 225/1148), which was consistent with previous studies (Xu et al., 2020; Oh et al., 2021). POD incidence in the SCD group (29.3%, *n* = 120/409) was significantly higher than that in the NSCD group (14.2%, *n* = 105/739). The results of the regression analysis and sensitivity analysis models supported the observation that hazard attributes of SCD were more likely to induce POD, proving the results of our preliminary study (Lin et al., 2021).

Aβ and tau play an important role in neurocognitive-related diseases (Bloom, 2014; Chandra et al., 2019). Aβ, a type of polypeptide that is hydrolyzed from amyloid precursor protein using β and γ secretory enzymes in a cascade, participate in the pathological progress of amyloidosis because of misfolding of the extracellular protein accumulated in senile plaques, and intraneuronal Aβ_42_ accumulation is more common than Aβ_40_ among them(Chen et al., 2017; John and Reddy, 2021). In contrast, one of the main functions of tau, a microtubule-associated protein, is to maintain the stability of axon microtubules; however, its hyperphosphorylated form hampers the normal function of neurons by twisting itself into tangles (Avila et al., 2004). Toxic Aβ peptides cause abnormal tau phosphorylation, transform into neurofibrillary tangles, and then lead to mitochondrial damage and calcium dysregulation, ultimately causing neuronal apoptosis and cognitive damage (Hussain et al., 2014). In this study, the intergroup comparison showed detectable and noteworthy differences in the CSF biomarkers (Aβ_42_, P-tau, and T-tau) between the patients with SCD and healthy individuals. Thus, we can reasonably presume that pathological biomarker burden also mediates the association between SCD and POD. In this study, the risk of patients suffering from POD increased with the increasing P-tau levels in CSF. A former study had shown that an increase in P-tau level was positively correlated with the number of abnormal tangles in the brain, and reduced Aβ_42_ concentration in CSF reflects its accumulation in amyloid plaques, altogether forming a core pathology of POD (Idland et al., 2017). However, no corresponding Aβ_42_ change was observed in the present study, this may be the result that P-tau rather than Aβ_42_ plays a dominant role in SCD-mediated POD pathology.

Furthermore, a previous study has shown that with the declination in cognitive function, T-tau and P-tau levels in CSF also decreased in patients with POD (Leoni et al., 2013). Naturally, CSF biomarkers could be used as one of the diagnostic indicators of SCD when other potential independent causative factors were excluded. Based on available data and evidence, we speculate that CSF biomarkers are a vital link between SCD and POD. Additionally, we first estimated that the CSF biomarker P-tau could partially mediate the effects of SCD on POD with a proportion of mediation (proportion = 31.25%, IE = 4.279 × 10^−2^, *p* < 0.001).

Cognition refers to any cognitive domain and is not confined to one point. Nevertheless, at present, most of the clinical measurements of SCD, including the questionnaire (SCDS) used in this study, mainly focused on the specific memory declination in patients (Jessen et al., 2014). However, a few studies have examined the risk factors for SCD, an early clinical syndrome. Therefore, in the present study, considering SCD as a dependent variable, we explored factors affecting SCD in the collected data by logistic analysis and sensitivity analysis. As expected, the incidence rate of SCD was strongly associated with age, and the recession pattern was practically similar to the POD mechanism (Lee and Lim, 2020). As mentioned earlier, SCD is conceptually independent of performance on a cognitive test (Jessen et al., 2014). The T-test results of MMSE and MoCA supported the observation that no significant cognitive difference was observed between the two groups in this study; however, the participants in the SCD group had higher preoperative PSQI scores. In other words, a sleep disorder may be associated with SCD; one reason behind the result is that good sleep can ensure the integrity of the central environment by maintaining the normal circadian rhythm and melatonin secretion (Ags/Nia Delirium Conference Writing Group, P.C., and Faculty, 2015). Moreover, CSF biomarkers can be used as important auxiliary diagnostic and predictive factors for SCD. The logistic regression and sensitivity analysis in this study showed that the increase in P-tau concentration was also the risk factor for SCD. Aβ_42_ is the best marker to predict the progression of patients from healthy aging to AD dementia, with T-tau and/or P-tau being the second choice (Lin et al., 2019). However, in this study, the multivariate logistic regression for Aβ_42_ showed no noteworthy result.

The present study has some limitations. First, despite we have included more well-qualified patients than those in the previous study, it was still limited to a single-center cohort study. Hence, more detailed analyses and experiments should be performed in multicenter studies for more reliable results. Second, associations among SCD, CSF biomarkers, and POD were examined in a cross-sectional design, meaning that we cannot dynamically monitor the occurrence and development of SCD and the fluctuation of biomarkers throughout the perioperative period in a single patient. Finally, considering the single source of SCD- or POD-related biomarkers in this study, in the future, multimodal neuroimaging techniques combing positron emission tomography and magnetic resonance imaging will help to further validate the study results.

To summarize, the present study indicated that patients with SCD are more likely to develop POD after undergoing total hip replacement. Moreover, SCD may mediate POD occurrence *via* P-tau. Hence, we recommend eliminating unhealthy lifestyles, as well as the early prevention and identification of SCD to reduce POD incidence.

## Data availability statement

The original contributions presented in the study are included in the article/Supplementary material; further inquiries can be directed to the corresponding author.

## Ethics statement

The studies involving human participants were reviewed and approved by the Ethical Committee Qingdao Municipal Hospital affiliated to Qingdao University, Qingdao, China. The patients/participants provided their written informed consent to participate in this study.

## Author contributions

FL contributed to the study design, data collection, statistical analysis, and manuscript preparation. XL was involved in surgical anesthesia. YL and XD were involved in the data collection. RD performed the neuropsychological testing. BW and YB contributed to the study concept and design, as well as manuscript preparation and review. All authors contributed to the article and approved the submitted version.

## Conflict of interest

The authors declare that the research was conducted in the absence of any commercial or financial relationships that could be construed as a potential conflict of interest.

## Publisher’s note

All claims expressed in this article are solely those of the authors and do not necessarily represent those of their affiliated organizations, or those of the publisher, the editors and the reviewers. Any product that may be evaluated in this article, or claim that may be made by its manufacturer, is not guaranteed or endorsed by the publisher.

## Supplementary material

The Supplementary Material for this article can be found online at: https://www.frontiersin.org/articles/10.3389/fnagi.2022.978297/full#supplementary-material
