# Supplementary material for: Subjective cognitive decline may mediate the occurrence of postoperative delirium by P-tau undergoing total hip replacement: The PNDABLE study
Source: Front Aging Neurosci. 2022 Nov 30;14:978297. doi: 10.3389/fnagi.2022.978297 (PMC9748689; doi:10.3389/fnagi.2022.978297)
Supplement: Supplementary file 2 [file Data_Sheet_2.docx]

**Subjective Cognitive Decline Scale（SCDS）**

**Part 1**

**Do you think that your memory is worse than memory of the past? If so, do you worry about that?**

**A.** No

**B.** Yes, but I don’t worry about that.

**C.** Yes, and I worry about that.

(Note: If the respondent chooses the B/C, we will go on the Part 2, or we will turn to Part 3.

**Part 2**

**Q1: How long has this feeling (SCD) been lasted?** (__years)

**Q2: How old are you when you had this feeling?** (Note: Q2 can be reckoned according to Q1 and patient's age)

**Q3: Besides memory, which of the following problems do you feel you have?** (Language / word choosing difficulties, organizational / planning skills, attention / concentration, etc. the investigator can give several specific examples) (Yes/No, if choose Yes, please explain specific.)

**Q4: Do you think your memory is worse than that of your peers?** (Yes / No)

**Q5: Is this feeling confirmed by the relevant insiders(need to enquire other family members)?** (Yes / No / Unclear)

**Part 3 （**A=2；B=1；C=0：Total points 12**）**

**Guide words**: Now I want to ask you some questions and understand your situation more.

**Q1: Compared with the past, do you feel that your memory of recent events has decreased recently?**

**A.** Yes, much worse than before.

**B.** Yes, a little worse than before.

**C.** No, is the same as before.

**Q2: Compared with the past, do you feel that you are unable to remember where you put things more often recently?**

**A.** Yes, much worse than before.

**B.** Yes, a little worse than before.

**C.** No, is the same as before.

**Q3: Compared with the past, do you find it difficult to recall the conversation 3-5 days ago recently?**

**A.** Yes, much worse than before.

**B.** Yes, a little worse than before.

**C.** No, is the same as before.

**Q4: Compared with the past, do you feel that it is difficult to remember what you have arranged to do recently?**

**A.** Yes, much worse than before.

**B.** Yes, a little worse than before.

**C.** No, is the same as before.

**Q5: Do the following situations happen frequently? —— It’s hard to remember your important days, such as your birthday.**

**A.** Frequently **B.** [Occasionally](http://www.baidu.com/link?url=h9yS2on4XKCgM8hzqh7moX5BcNlYGYsYu_vy2kM74CAkMEoexzDMWmbLM7UHjE26dqg0Le4DqyalLR2PkuBcLDVibz0PfJI_s94_pygG5g3) **C.** Never

**Q6: Do the following problems happen frequently? Forget the phone number that you use frequently.**

**A.** Frequently **B.** [Occasionally](http://www.baidu.com/link?url=h9yS2on4XKCgM8hzqh7moX5BcNlYGYsYu_vy2kM74CAkMEoexzDMWmbLM7UHjE26dqg0Le4DqyalLR2PkuBcLDVibz0PfJI_s94_pygG5g3) **C.** Never

**Total score of the Part 3: _____points（0 ~ 12 points）**

**Part 4**

**Q1: Do you look for the right words more often than before?** 1. Yes; 0. No.

If so ,

Are you worried about that? 1. Yes; 0. No.

Do you feel that your ability is worse than your peers in this respect? 1. Yes; 0. No.

**Q2: Do you feel more difficult to plan or arrange things in an orderly way?** 1. Yes; 0. No.

If so ,

Are you worried about that? 1. Yes; 0. No.

Do you feel that your ability is worse than your peers in this respect? 1. Yes; 0. No.

**Part 5**

**Q1: Is SCD caused by mental disorders (such as depression and anxiety)?** (Yes / No)

**Q2: Can SCD be explained by other medical diseases?** (Yes / No)

**Q3: Is SCD caused by drugs or other substances abuse?** (Yes / No)

**SCDS instructions**

The questionnaire design is based on the SCD-I recommendation [1,2], with the hospital environment as the research background, aim to the memory function of the respondents, setting two SCD evaluation methods: classification index (the first part) and continuous index (the third part). At the same time, the second part is set as a supplement according to SCD plus criteria [1].

1) In part 1, there is only one question: the person who chooses C is considered as the basic condition of SCD; in addition, other conditions for final judgment of SCD include: the scores of MMSE, MOCA and ADL are normal, and other neuropsychiatric diseases (except AD), medical disorders, drugs or other substances abuse are excluded (the part 4).

2) In part 2, five questions were added to help judge the possibility of preclinical AD;

3) The part 3 adopts Likert scale and top 9 SCD items [3] to adapt the subjective memory decline scale [4]. After the adaptation, six questions are set, with 0-2 points for each question and the highest score is 14 points.

4) The part 4 is designed for other cognitive domains (Language and executive function) besides memory.

5) The part 5 is set for the exclusion criteria: excluding SCD caused by other neuropsychiatric diseases (except AD), medical disorders, drugs or other substances abuse. The subjective description of the respondents and the objective score of the relevant scales were comprehensively judged by the investigators.

6) The parts 1and 3 should be filled in by the patients themselves; the part 2, 4 and 5 should be filled in by the investigators.
